# First, Do No Harm: Why Philanthropy Needs to Re-Examine Its Role in Reproductive Equity and Racial Justice

**DOI:** 10.1089/heq.2019.0094

**Published:** 2020-03-12

**Authors:** Karen A. Scott, Stephanie Bray, Monica R. McLemore

**Affiliations:** ^1^ Obstetrics, Gynecology, and Reproductive Sciences Department, School of Medicine, University of California, San Francisco, San Francisco, California.; ^2^ United Way California Capital Region, Sacramento, California.; ^3^ Family Health Care Nursing Department, School of Nursing, University of California, San Francisco, San Francisco, California.

**Keywords:** philanthropy, reproductive equity, racial justice, cultural rigor

## Abstract

The philanthropic-industrial complex fosters the belief that the most marginalized communities lack an existing repository of historical and contemporary knowledge to address social and health inequities. In so doing, philanthropy has contributed to the diminishing political power, legitimacy, and effectiveness of community voices and leadership in reproductive equity through research injustice, cultural arrogance, philanthropic redlining, and community harm. Black Feminism and Reproductive Justice, as mutually aligned theories and praxes, are described as new ethical standards for philanthropic accountability. Funders must embody the equity they aspire to see and build through the operationalization of cultural rigor to advance structural equity and racial justice and to sustain community engagement in research.

## Introduction

Philanthropy has long been the driver of the greater good through the investment of private assets from a position of morality, emotions, and charity. One need only examine the proliferation of philanthropies during a 30-year period between the 19th and 20th centuries. Whether foundations that funded the education of free Black people after slavery, or those that helped build the social safety net, including health care, all became part of a philanthropic industrial complex that exists to this day.^1^ This complex continues to foster cultural arrogance and the belief that the most marginalized communities lack an existing repository of historical and contemporary knowledge to address social and health inequities. In so doing, philanthropy has contributed to the diminishing political power, legitimacy, and effectiveness of community voices and leadership through research injustice.^2^ The current funding of research on the Black reproductive health crisis is clear evidence of this.

Obstetric violence and mistreatment have been globally documented across the world.^3^ Reproductive injustice has also been shown at the individual, community, and structural levels.^4^ Clinical and public health research have provided crucial data that describes these problems, yet a lack of data exists about the role that funders, including local, state, and federal government as well as philanthropists, play in resolving health inequities through structural equity and racial justice.^5^ A glossary of terms and definitions are included (Table 1) to support the reader's understanding and interpretation of the material and concepts described in the analysis.

**Table 1.** Glossary of Terms

Terminology	Definition
Black birth workforce	Black people who support birth and birthing people along with attending births, within and outside of the hospital settings, regardless of compensation, education, training, or discipline (i.e., birth educator, lactation educators, prenatal fitness/yoga instructors, labor and delivery nurses, midwives, doulas, physicians, healers, therapists, and advocates)
Black feminism	Patricia Hill Collins described four themes of Black feminist theory in 1990, all stemming from a Black woman's standpoint in 1989: (1) Black women empower themselves by creating self-definitions and self-valuations that enable them to establish positive, multiple images and to repel negative, controlling representations of Black womanhood; (2) Black women confront and dismantle the “overarching” and “interlocking” structure of domination in terms of race, class, and gender oppression; (3) Black women intertwine intellectual thought and political activism; and (4) Black women recognize a distinct cultural heritage that gives them the energy and skills to resist and transform daily discrimination. As a praxis, that Black feminism in research requires leadership as well as knowledge construction and replication about the phenomenon of pregnancy, labor, birth, and immediate postpartum as much as possible from women of color in general or Black women, in particular.^4^
RJ	As a theory, practice, strategy, and public health praxis, RJ is grounded in four principles: (1) every person has the right to decide if and when to become pregnant and to determine the conditions under which they will birth; (2) every person has the right to decide they will not become pregnant or have a baby and have options for preventing or ending pregnancy that are accessible, approachable, acceptable, available and accommodating, affordable, and appropriate; (3) individuals have the right to parent children they already have with dignity and with the necessary social supports in safe environments and healthy communities without fear of violence from individuals or the government; and (4) individuals have the right to disassociate sex from reproduction and that healthy sexuality and pleasure are essential components to whole and full human life. Importantly, RJ is a change in language, meaning, and inclusion that was led, and must continue to be led, by Black, indigenous women/people, and women/people of color^7^
Research justice	As defined by Andrew J. Jolivétte, research justice is a strategic framework and methodological intervention that seeks to transform structural inequities in research based on three core actions: (1) values and amplifies three forms of knowledge: cultural and spiritual, mainstream, and experiential knowledge, for the purposes of achieving equal political power and legitimacy for, by, and with community; (2) examines the relationships and intersections between research, knowledge construction, and political power/legitimacy; and (3) centers community experts as vital partners in a movement-building strategy in three areas: knowledge construction and self-determination, community mobilization, and social transformation and policy reform^2^

RJ, reproductive justice.

If we are to address this crisis with radical curiosity and courage, the philanthropic sector should re-examine its role in promoting a status quo that prioritizes a limited set of disciplinary and demographic expertise to a chosen few at the exclusion and erasure of Black community members, activists, artists, and scholars. Specifically, the philanthropic sector's inability to prioritize ongoing examinations of birthing while Black in the United States through the expertise, experience, and filter of Black women scholars underscores a naivete or overt defiance to fully accept and appreciate how dynamic and multiple identities and positionalities of Black women open them up to simultaneous, overlapping, and connected oppressive and exploitative structures, systems, and people. Black Feminism and Reproductive Justice as mutually aligned theories and praxes offer a road map to the types of assumptions, conceptualizations, methodologies, goals, interventions, values, and positionality that are necessary in the health services research and the translation of science into practice for, by, and with Black community, content, and patient experts.^6,7^

In other words, projects deemed “fundable” and not necessarily culturally relevant and responsive to community identified needs often lack epistemological diversity, disruptive innovation, equitable transformation, and radical care. The care is designated as radical simply due to the ongoing lack of acknowledgment and protection of the dignity, sanctity, and humanity of Blackness in health services research and provision. Thus, radical care, at its core, is grounded in trusting Black women and people to be the architects, engineers, and providers of care that Black mothers and birthing people deserve, as defined through the theories and praxes of Black Feminism and Reproductive Justice. At the same time, the philanthropic sector would benefit from examining funding priorities, goals, and mechanisms in relationship to the power and potential for communities to drive knowledge construction, self-determination, community mobilization, and social transformation and policy reform, as defined research justice methodologies for social change.^8–10^

Community harm then becomes the byproduct of philanthropic redlining, a discriminatory practice of inequitable distribution of philanthropic funds combined with neglect of justice-centered Black-led institutions, fueled by discriminatory notions that Black-led institutions are ineffective, inferior, and fraudulent. The consequence of historical philanthropic redlining is that as little as 8% of foundation funding goes to people of color, and 1% to the Black community, reinforcing the belief and practice that social change within the Black community must be led by white-governed institutions.^11^ Contemporary philanthropic redlining results in funding Black-led teams under the conditions of white expediency and white urgency. White expediency is the quality of being convenient and practical despite possibly being improper or immoral, resulting in differential risks, barriers, burdens, and benefits. White urgency is the quality of being important that requires swift action that advantages white people and those proximal to whiteness while simultaneously disadvantaging and harming Black people and those amplifying Blackness. Contemporary philanthropic redlining further harms Black-led institutions by requiring them to do the most, in dismantling racist systems and structures, with the least amount of resources in a short amount of time, while simultaneously seeking additional consultation from white/non-Black men of color or white/non-Black men of color teams who lack the expertise to lead the work, but hold enough value to the funder to remain engaged in the work.

Cultural arrogance, philanthropic redlining, and community harm create a toxic stew of assumptions that shape research questions and research agendas. This stew feeds three common misconceptions based on a hierarchy of knowledge construction and innovation that devalues the expertise of Black birthing people and the scholarship of Black women scholars that philanthropists must acknowledge: (1) Only white-governed institutions possess the scientific rigor, integrity, and expertise to conduct research meaning that the innovations within the community are of lesser value than those brought forward by majority institutions; (2) clinicians and data scientists are the only ones qualified to drive innovations in health services research and quality improvement initiatives; and (3) the solutions to reducing reproductive mortality and morbidity do not already exist within the community that carries the greatest burden. These three misconceptions not only dismiss cultural humility as essential but also turn a blind eye to the importance of cultural rigor in the science of health equity and community engagement. We use these three misconceptions with exemplars to inform a call to action that provides short- and long-term solutions to leverage philanthropy and its role in catalyzing advancement toward health equity. However, we must first define cultural humility and rigor.

## Defining Cultural Humility

Two Black women physicians and public health scholars, Melanie Tervalon, MD, MPH, and Jann Murray-Garcia, MD, MPH, first defined the tenets of cultural humility in 1998: (1) lifelong commitment to self-learning and critical self-reflection; (2) dismantling inherent power imbalances and building respectful relationships between patients and clinicians; (3) developing mutually beneficial non-hierarchical clinical and advocacy partnerships with communities; and (4) creating institutional alignment and accountability.^12^ The first three of these tenets are individually focused; however, we believe an expansion of the fourth is necessary to understand how institutions and organizations can enact cultural humility.

Given the alarming and disparate numbers of Black birthing people continuously dying and nearly dying under the care of physicians in U.S. hospitals during labor, birth, and postpartum—despite access to reproductive technology and quality improvement toolkits—we feel a sense of immediacy to emphasize that Black Mamas Matters and to support and sustain living and thriving when birthing while Black.^13^ First, funders and organizations should focus their attention to set group hires as a standard in achieving health equity.^14^ Any single minoritized individual is subject to harm—including denial of their expertise and the unrealistic expectation to shift an organization toward justice/equity without institutional power to achieve this goal. Next, funders and organizations need accountability structures built with communities beyond advisement—community members need the power to make decisions, guide the development of humans, money, and time resources, and call for a pause on work when necessary to address both the content and the process of the work. Finally, funders and organizations need ethical conflict resolutions strategy and principles of partnership before any initiation of work.

In the absence of cultural humility, cultural arrogance propagates itself, invading institutions as an insidious and diabolical force, shaping workforce characteristics, compositions, competencies, and compensations through immoral, unethical, and unjust truths and mechanisms. This cultural arrogance establishes and perpetuates the supremacy of empirical knowledge above historical, cultural, spiritual, mainstream, relational, and experiential knowledge of marginalized communities. Institutional cultural arrogance advances a dominant group while simultaneously silencing and sabotaging the most relevant and radically imaginative voices needed to disrupt and (re)design health care and public health systems.

## Defining Cultural Rigor

Cultural rigor expands the value of cultural humility beyond interprofessional education and health services provisions. Cultural rigor offers four modalities for institutional recognition and mitigation of cultural arrogance, philanthropic redlining, and community harm. As a social movement, cultural rigor dismantles the lies and starves the delusions that Black women's scholarship, Black birth workforce, and Black tech lack the scientific rigor to align clinical, structural, and social determinants of health, and design robust and reproducible measures and interventions. Cultural rigor creates a shift in the language, assumptions, priorities, and mechanisms utilized in philanthropy to achieve health equity and community engagement.

Likewise, cultural rigor acknowledges and advances the humanity, value, and social worth of Black mothers, birthing people, women scholars, artists, and activists. As an analytic framework, cultural rigor grounds the scientific method and philanthropic giving within the four tenets of cultural humility. Scientific rigor as defined by the National Institutes of Health (NIH) is “the strict application of the scientific method to ensure robust and unbiased experimental design, methodology, analysis, interpretation and reporting of results.”^15^ Scientific rigor is necessary for increasing the replicability of study methods and generalizability of study findings. However, the absence of cultural rigor as part of the overall rigor can lead to the conduct of research with statistically significant results that lack clarity and cultural relevance to community identified research priorities, in comparison to the research questions and aims identified by scientists and funders without community participation and approval. Cultural rigor demands that the researched serve as the unfettered architects, incubators, and accelerators of their own radical imaginations, connections, and destinies. Maintaining fidelity to the model of cultural rigor requires transfer of power from the researchers and funders to the researched^16^ and continuously examines and closes the gaps between the current ethics and aspirations of funders, scientists, impacted communities, and activists.^17^

As a praxis, cultural rigor mandates the operationalization of Black feminism, reproductive justice, and research justice, along with participatory data and technology development and dissemination in health care services provision, research, quality improvement, and policy. Cultural rigor ultimately asserts that all white-governed institutions cannot serve as the benefactors, oppressors, and redeemers of Black women's genius, and it reinforces an inconvenient truth: You have no answer that Black women don't already possess.^18^ Cultural rigor requires funders to divest from the comfortable, convenient, common, and complacent practices of tokenism, decoration, manipulation, and exploitation, which reinforce a hierarchy of knowledge generators and systems disruptors.^19^

As a vision for racial justice and health equity, cultural rigor creates optimal conditions and opportunities for engaging the full and prime potential and power of individuals, communities, and systems, where radical imagination, innovation, and care thrive, and voices, values, and beliefs are aligned, integrated, and reflected back to the community. Further, cultural rigor breaks down silos and challenges the status quo through radical truth telling, transparency, reconciliation, and healing for all parties involved. Funders are strategically positioned to model the change they aspire to see in society across sectors, silos, and systems by dismantling the notions by which whiteness and all white-governed institutions define and drive scientific innovations and disruptions. By grounding philanthropy in the science of cultural rigor, funders can then radically reimagine public and community inclusion in a tiered approach, moving from engagement to consultation, involvement, and then to partnerships.^19^

## Addressing Misconceptions

### White-governed institutions

The unfortunate reality of health services provision, education, policy, and funders is that these domains are primarily white-governed institutions. Adding to this problem is the immature understanding of racialized dynamics within how humans, money, and time resources are allocated to resolve disparities and achieve health equity. Diversification of the health care workforce is the priority that needs to be addressed to populate all of these domains that should provide community wisdom and expertise.

### Clinicians and data scientists

Clinicians and data scientists have a significant responsibility to understand the unintended consequences of their research findings and dissemination to not perpetuate or maintain individual or mother blame across the reproductive spectrum. In addition, it is important to have appropriate community engagement and oversight of research to ensure the reduction of harm and expansion of effective and timely interventions.

### Where the solutions to reducing reproductive mortality and morbidity reside

Effective interventions that will lead to the resolution of health inequities reside in the people and communities who experience the greatest burden. Research, funding, policies, and education must center on those most impacted to ensure appropriate short-term, interim, and long-term metrics for success and innovation.

## Call to Action: Advancing Cultural Rigor in Philanthropy

Therefore, we call on funders to adhere to new standards for achieving and sustaining institutional alignment and accountability to patients, the public, providers, leaders, and learners in racial equity and reproductive justice. First, we recommend that funders conduct their own internal racial justice and reproductive health equity readiness assessment, before the dissemination of funding priorities, expectations, and money. Funders can benefit from a critical 360° examination of the role and impact of their own organizational characteristics, workforce competencies, culture, and operating mechanisms that either obstruct or advance Black-centered racial justice and reproductive health equity. Although it is important to acknowledge the wide variation of grantmakers and philanthropists currently funding organizations and institutions addressing racial equity and reproductive justice, it is past time for the development of a new standard that radically transforms grantmaking by centering on the wisdom, assets, and leadership of communities that bear the greatest burden of reproductive harm and oppression and not only those organizations and institutions that purport to know best what communities need.

Second, funders, scientists, clinicians, payers, policy makers, and community leaders can benefit from an anti-racist process evaluation of existing philanthropic policies, practices, and programs that perpetuate the exclusion, erasure, or erosion of the lived experiences, knowledge, and scholarship of Black women scientists, Black women-led community-based organizations (CBOs), and Black mothers and birthing people. Identifying areas of alignment and disconnect between the program design, inputs, activities, and outputs as determined by, for, and with impacted communities, funders, and grantees in an anti-racist process evaluation strengthens funder alignment and accountability to the needs and priorities of the most impacted communities. Consequently, both funders and grantees would then have to confront the hidden truths about their individual and collective perceptions, knowledge, relationship to, and understanding of race, racism, and the racialization of the reproductive outcome under examination.

Third, we recommend that both funders and their grantees actively commit their power, money, and time to creating and sustaining a diverse and inclusive research team with equitable representation of the communities carrying the greatest burden of death, near deaths, and intersectional oppression and violence. As a result, budget line items and narratives would reflect allocation of money and resources to community leaders, experts, and activities that align with the prevalence and impact of the disparate reproductive outcome of interest. Further, the (re)distribution of power, money, and time to Black women-led CBOs serves as a primary disruption of the common practice of funding all white-governed institutions and catalyzes workforce diversification and development.^20^ Institutions that hire only one Black person/Black woman scholar to undo the trans-generational effects of more than 400 years of structural, institutional, and interpersonal oppression, exploitation, violence, and harm are irresponsible, immoral, and unethical.

The simultaneous implementation and embodiment of cultural rigor in all four modalities is the community-based institutional review board (IRB).^21^ Community-based IRBs would examine institutional alignment and accountability to racial justice and reproductive health equity based on community-defined and -driven priorities, milestones, and timelines. Community-based IRBs would also examine the cultural rigor in the ethical, theoretical, methodological, and dissemination approaches proposed in the design of the research team and study. In so doing, the operationalization of community-based IRBs would support and fuel the radical reimagination of community self-determination through participatory technologies and predictive analytics. Moreover, funding of sustainable community-based IRBs would be a very practical and compelling strategy to facilitate the political power, legitimacy, and effectiveness of community voices and leadership through research justice.

## Conclusion

We invite funders to fully embrace and practice cultural rigor as they expand their portfolios to address reproductive equity in the United States. To create different partnerships and achieve different outcomes, funders must undergo the process of decolonizing their philosophies, priorities, practices, processes, power structures, and people in leadership. Funders must embody the equity they aspire to see by operationalizing cultural rigor in the science of advancing structural equity and sustaining community engagement.

## Abbreviations Used

CBO: community-based organization
IRB: institutional review board
NIH: National Institutes of Health
RJ: reproductive justice
